# Anthropogenic Particle Ingestion in the Starry Round Ray *Urotrygon asterias*: High Occurrence Independent of Biological and Spatial Predictors

**DOI:** 10.1155/sci5/1691340

**Published:** 2026-10-29

**Authors:** Allan Rosales-Valencia, Felipe Amezcua, Carlos René Green-Ruíz

**Affiliations:** ^1^ Posgrado en Ciencias del Mar y Limnología Universidad Nacional Autónoma de México Ciudad de México Mexico unam.mx; ^2^ Instituto de Ciencias del Mar y Limnología Universidad Nacional Autónoma de México Sinaloa Mexico unam.mx

**Keywords:** µFTIR–ATR spectroscopy, batoids, bycatch, cellulose fibers, Gulf of California, microfibers, microplastics

## Abstract

The ingestion of anthropogenic particles by marine organisms is now widely reported, yet rays and skates remain poorly studied, especially in tropical regions. Particle ingestion was examined in the starry round ray (*Urotrygon asterias*), a common bycatch species found in the Gulf of California shrimp fishery. We analyzed the digestive tracts of 114 individuals collected in July and August 2019 to determine how frequently particles were present, how many were ingested, and what materials they were made of using µFTIR–ATR spectroscopy. We also tested whether sex, body size, diet, or capture zone influenced ingestion patterns. Anthropogenic particles were found in 82% of the rays, with a mean of 2.35 ± 2.21 particles per individual examined (*n* = 114). Almost all particles were fibers (98.9%). Natural fibers dominated, with cellulose and cotton together accounting for 84% of all particles, followed by synthetic polymers (13.4%, mainly polyester) and semisynthetic rayon (2.2%). Particle abundance and particle composition did not differ between males and females across size classes and among capture zones, and no clear association with diet composition was observed. Although diet varied among regions, particle ingestion patterns did not differ significantly by region. These results suggest that particle ingestion in this species may be consistent across the biological and spatial groups evaluated, although unbalanced sample sizes or unmeasured environmental factors may have limited the ability to detect real differences. The dominance of textile‐related fibers points to everyday human activities as a possible source of exposure in this coastal system, though their specific origin would require confirmation through direct source‐tracking analysis. This study provides the first record of anthropogenic particle ingestion in the genus *Urotrygon* and offers baseline information for the Gulf of California.

## 1. Introduction

Plastic pollution has become a persistent and widespread problem in marine ecosystems, fueled by the steady rise in global plastic production, which reached 413.8 million tons in 2023 [1]. Once in the ocean—through rivers, wastewater, urban runoff, or direct dumping—plastic debris breaks down into small particles less than 5 mm in size because of sunlight, physical abrasion, and biological processes [2–4]. These particles enter marine food webs in several ways: Organisms may ingest them directly, consume contaminated prey, or encounter them in sediments. Exposure depends on where species live and feed, how particles are distributed in the environment, and the physical characteristics of the particles themselves [5, 6]. Microplastics are not evenly spread across the ocean; they tend to accumulate in coastal waters, semienclosed seas, and subtropical gyres, creating uneven patterns of exposure among marine organisms [7, 8].

Microplastics have been defined as “Any synthetic solid particle or polymeric matrix, with regular or irregular shape and with size ranging from 1 μm to 5 mm, of either primary or secondary manufacturing origin, which are insoluble in water” [9]. During early studies on microplastic contamination, many observed particles were classified solely based on their morphology, which may have led to an overestimation of microplastic abundance by inadvertently including natural fibers [10]. The routine use of spectroscopic techniques, such as µFTIR and Raman, has demonstrated that a substantial proportion of recovered particles corresponds to natural fibers, primarily cellulose and cotton [10].

In this context, several authors have proposed the term anthropogenic particles, which encompasses both synthetic polymers and natural fibers of anthropogenic origin that have undergone industrial processing, including dyeing, bleaching, and the incorporation of chemical additives [11, 12]. Although natural fibers are generally more biodegradable than synthetic polymers, industrial processing and associated chemical additives may alter their properties and pose potential risks to aquatic organisms [10].

Experimental evidence indicates that both synthetic and natural fibers can induce biological effects; however, there is currently no consensus regarding which type poses the greater ecological risk. Some studies have reported no significant differences in growth, immune response, or development of fish exposed to polyester and cotton fibers [13], whereas others have observed higher mortality in organisms exposed to synthetic fibers [14] or digestive and immunological alterations associated with natural fibers [15]. Furthermore, recent studies suggest that toxicity depends not only on the chemical composition of the fibers but also on their physical characteristics, as well as associated additives and adsorbed contaminants [16]. These findings highlight the importance of considering both synthetic particles and industrially processed natural fibers when assessing anthropogenic particle contamination in marine ecosystems.

During sample processing, there is a risk of contamination by anthropogenic particles, particularly airborne fibers, which may lead to an overestimation of the particles present in biological samples if appropriate quality assurance and quality control (QA/QC) measures are not implemented [17]. Therefore, it is essential to apply protocols aimed at minimizing secondary contamination and assessing its potential influence through procedural controls. Recommended practices include washing and rinsing laboratory equipment with Milli‐Q water, wearing laboratory clothing and coats made from natural fibers (100% cotton) to reduce the release of synthetic fibers, and including procedural blanks throughout sample processing [17].

In addition to these quality control measures, methodological standardization remains one of the main challenges in anthropogenic particle research because factors such as digestion procedures, filtration systems, filter type, and QA/QC measures can influence particle recovery and identification [18]. These considerations are particularly relevant for elasmobranchs, whose gastrointestinal tissues are characterized by a high lipid content and dense connective tissue, requiring digestion protocols specifically designed to maximize particle recovery without compromising particle integrity during sample processing. In this regard, Cruz‐García & Amezcua [19] developed and validated a protocol specifically for elasmobranch gastrointestinal tissues, achieving recovery efficiencies exceeding 90% through a sequential KOH–H_2_O_2_ digestion protocol and validating the method using positive controls and procedural blanks.

Sharks and rays are increasingly exposed to anthropogenic particles, although information remains limited across many regions and species. Studies have reported high ingestion frequencies in several shark species. For example, demersal sharks from U.K. waters showed a 67% occurrence of cellulose, polypropylene, and polyester particles [6], whereas all examined tiger sharks contained anthropogenic particles, suggesting contamination through prey [20]. Similarly, 79.3% of juvenile scalloped hammerheads from the Gulf of California contained cellulose particles [21]. Despite these findings, information on anthropogenic particle ingestion in rays and skates, particularly benthic‐feeding species, remains scarce. The few available studies have reported different types of anthropogenic particles in species such as Haller’s round ray, the longnose stingray, and the spiny butterfly ray [22, 23]. Recent studies have also consistently indicated a predominance of anthropogenic natural fibers over synthetic polymers in the gastrointestinal tracts of various elasmobranch species [6, 24]. However, the factors determining the ingestion and retention of such particles are not yet well understood.

Despite its abundance and ecological importance, studies on the starry round ray do not exist. Populations of this species occurring in the Gulf of California were previously identified as *Urotrygon chilensis*. However, a recent taxonomic revision based on morphological and genetic evidence demonstrated that the northern morphotype previously assigned to *U. chilensis* corresponds to *Urotrygon asterias*, which is now recognized as a valid species distinct from *U. chilensis* [25]. This taxonomic assignment has subsequently been adopted in studies conducted in the southeastern Gulf of California, including populations from the coast of Sinaloa [26]. Therefore, the name *U. asterias* is adopted for the specimens examined in the present study. This species primarily feeds on small invertebrates like polychaetes, crustaceans, and mollusks, which exposes it to sediment where particles can accumulate. Contamination patterns are influenced by biological traits and ecological factors, with feeding strategy and habitat playing significant roles. Species that feed on or within sediments are more likely to encounter fibers that settle on the seafloor, while larger predators may accumulate particles through trophic transfer [5, 20]. Studies on the relationship between body size, life stage, and particle ingestion have shown inconsistent results [6]. Differences between males and females are minimal, and the impact of maturity is still unclear. Most research on anthropogenic particle ingestion has focused on sharks, leaving rays and skates relatively understudied despite their unique ecological characteristics.

Key gaps in contamination studies include the underrepresentation of rays and skates, limited evaluation of bycatch species for additional stressors, and the prevalence of cellulosic fibers alongside plastics. Understanding how biological traits and ecological factors influence contamination patterns in batoids and the importance of species‐specific differences in widespread contamination areas remains unclear. Despite its ecological significance, the Gulf of California lacks comprehensive baseline information.

This study is the first to assess anthropogenic particle ingestion in the starry round ray from the Gulf of California. We utilized spectroscopic analysis to ascertain the frequency, ingested quantity, and material composition of particles within the digestive tract. The study also investigated whether ingestion patterns varied based on biological traits (sex and body size) or ecological factors such as diet composition and capture zone. The hypothesis was that larger individuals would have more particles due to cumulative intake. We also expected that if exposure was widespread and uniform across the region, ingestion patterns would not strongly associate with biological or spatial factors. The study compared its findings with existing data on anthropogenic particle ingestion in other elasmobranch species from the Gulf. By filling a taxonomic and regional gap, this study offers essential baseline information on a common bycatch species and enhances understanding of anthropogenic particle exposure in the coastal ecosystems of the Gulf of California.

## 2. Materials and Methods

### 2.1. Sample Collection

Specimens of the starry round ray were collected from the coastal zone of Sinaloa, Mexico, during demersal shrimp fishery surveys in July and August 2019. The coastline was divided into three regions—north, central, and south—following the Council for Economic Development of Sinaloa (CODESIN) classification (Figure 1). All captured rays were frozen immediately on board and transported to the laboratory.

**FIGURE 1:**
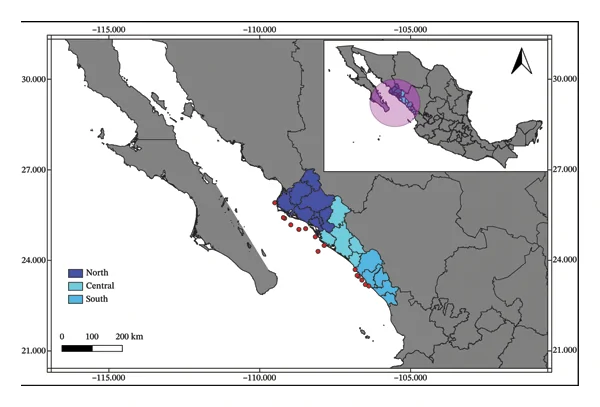
Sampling stations (red dots) in three distinct regions of the state of Sinaloa (north, central, and south) from the southeastern Gulf of California.

### 2.2. Biological Data and Dissection

In the laboratory, each specimen was measured (disc width, DW) and sexed based on the presence or absence of claspers. The digestive tract, from the esophagus to the intestines, was removed using sterilized instruments. Stomachs were carefully opened, rinsed with Milli‐Q water, and examined under a stereomicroscope to identify prey to the lowest possible taxonomic level. Stomach content analysis focused on rapid processing to minimize environmental contamination.

### 2.3. Quality Control

Strict contamination control procedures governed all laboratory work. Before processing any samples, all glassware, metal instruments, filtration units, and work surfaces were thoroughly washed, rinsed with filtered water, and kept covered until use. Whenever possible, dissections and subsequent handling were conducted under clean‐air conditions (in a laminar‐flow cabinet), and samples were never left unnecessarily exposed. To minimize airborne and clothing‐derived fibers, only natural‐fiber lab coats were worn, and synthetic garments were avoided. The exterior of each specimen was carefully rinsed before dissection to remove any adhering particles. Procedural blanks (in triplicate) were included with every batch and processed alongside the samples using filtered water, allowing us to detect and account for any background contamination introduced through air, reagents, or equipment. In addition, positive controls were run in parallel by spiking clean tissue with a known number of anthropogenic particles of identified polymer type, enabling us to evaluate recovery efficiency throughout digestion and analysis.

Procedural blank controls (*n* = 36) contained a total of six anthropogenic particles throughout the entire sample processing procedure, corresponding to a mean of 0.17 particles per blank and representing only 2.24% of the 268 particles detected in biological samples. These consisted of four cellulose fibers, one cotton fiber, and one polyester fiber, ranging from 0.758 to 3.476 mm in length (Supporting Table S1). Although the materials identified in the procedural blanks were also detected in the biological samples, their extremely low abundance relative to the total number of recovered particles indicates that laboratory contamination had a negligible influence on the overall results. Munno et al. [27] recommend reporting sample particle counts together with the number of particles detected in procedural blanks, as the most appropriate procedures for this type of adjustment are still under development. Following this recommendation and considering the low level of laboratory contamination observed in our procedural blanks, no blank correction was applied to the data. Similar approaches have been adopted in previous studies where procedural blank contamination was considered negligible and therefore did not justify the application of blank correction [28–30].

Positive controls were included to independently assess recovery efficiency and material identification performance. Clean tissue was spiked with 12 fibers of known prespike material identity and color (eight black and four blue) and carried through the complete digestion, filtration, and µFTIR–ATR workflow. All 12 spiked fibers were recovered, corresponding to a recovery efficiency of 100%. Of the 12 recovered fibers, nine yielded successful µFTIR–ATR identifications (75% identification success), with a mean library match of 86.6% (range: 68%–97%). All nine successful identifications agreed with their known prespike material identities, whereas three cellulose fibers were recovered but did not yield a reliable µFTIR–ATR identification. Because the positive control consisted of a single spike batch, a standard deviation of recovery could not be estimated, and recovery is therefore reported from a single trial. The > 85% match threshold described above was applied to the classification of biological particles; for the positive controls, all available library matches are reported for validation purposes. Full perfiber results are provided in Supporting Table S2.

### 2.4. Digestion of Organic Material and Filtration

For each specimen, the entire gastrointestinal tract was examined to ensure a complete assessment of ingested particles. Posteriorly, they were digested according to the methods proposed by Hermsen [17], using a 20% potassium hydroxide (KOH) solution for 72 h at room temperature, avoiding heating to prevent potential polymer alteration. The digested material was vacuum‐filtered using 1.5 µm pore‐size paper membranes and handled with the same precautions described above to maintain sample integrity throughout the process. Filters were stored in covered glass Petri dishes until particle examination.

### 2.5. Particle Identification and µFTIR–ATR Analysis

Suspected anthropogenic particles were counted, photographed, and measured under a stereomicroscope (Zeiss Stemi 508) equipped with a digital camera (Zeiss AxioCam ERc5s). All observed particles were analyzed using µFTIR–ATR with a germanium crystal. Spectra (650–4000 cm^−1^, 4 cm^−1^ resolution) were matched against a reference library, and particles with > 85% match quality were classified by material type.

µFTIR–ATR spectroscopy confirms chemical composition but cannot by itself distinguish between processed textile‐derived cellulose fibers and naturally occurring environmental cellulose. However, all cellulose‐based particles identified in this study displayed distinct artificial coloration (predominantly blue and black), consistent with industrial dyeing processes rather than the translucent or pigment‐degraded appearance typical of environmental plant‐derived cellulose, and the cellulose fraction identified in this study is therefore considered predominantly of anthropogenic origin.

### 2.6. Statistical Analysis

Size cohorts were estimated using kernel density analysis [31], with the Sheather–Jones bandwidth selector as defined by Equation (1).

(1) f∧x=1nh ∑i=1nKx−Xih,

where *h* is the bandwidth and *K*(*x*) is the Gaussian kernel function. The analysis was performed using R software Version 4.4.1.

Dietary composition was quantified using frequency of occurrence (FO) [32], as defined by Equation (2).

(2) FO=nTS.100.

FO represents the frequency of occurrence, *n* is the number of stomachs containing a given species, and TS is the total number of stomachs that were analyzed.

Differences in particle abundance among zones, size classes, sexes, and dietary diversity were evaluated using a generalized linear model (GLM). Because anthropogenic particle abundance is a count variable, we retained the complete dataset (*n* = 114), including the 20 individuals with no detected particles, and modeled abundance with count‐based GLMs rather than excluding the zero counts. A Poisson GLM with a log link served as the baseline but showed clear overdispersion (Pearson dispersion = 2.02; variance‐to‐mean ratio = 2.07); we therefore fitted a negative binomial (NB) GLM, which relaxes the Poisson equidispersion assumption by estimating an additional dispersion parameter. To assess whether the zero counts arose from a process distinct from the count process, we also fitted a hurdle model (a binomial component for particle presence combined with a zero‐truncated NB component for abundance) and a zero‐inflated negative binomial (ZINB) model.

All models included size class (S, M, L), sex, sampling zone, and dietary diversity (Shannon–Wiener index) as predictors. Dietary diversity was represented by the Shannon–Wiener index because some prey categories were consumed by very few individuals, making analyses based on individual prey types unreliable; the index provided a continuous measure of dietary breadth per individual. Competing models were compared using the Akaike and Bayesian information criteria (AIC and BIC), and the contribution of each predictor in the selected model was evaluated with Type II likelihood‐ratio tests. To test the hypothesis of cumulative accumulation directly, DW was additionally modeled as a continuous predictor, and its relationship with particle abundance was examined with Spearman’s rank correlation. All analyses were performed in R; the NB model was fitted with the MASS package [33]. And the hurdle and zero‐inflated models with the pscl package [34].

We conducted two PERMANOVA analyses (9999 permutations, Bray–Curtis similarity, Type III sums of squares) to evaluate whether biological factors (sex and body size) and ecological factors (capture zone) influenced (1) anthropogenic particle ingestion patterns and (2) diet composition. Analyses were based on fourth‐root‐transformed abundance matrices to reduce the influence of dominant items. To accommodate samples with no detected items in the corresponding matrix, a dummy variable with a value of 1 was added to all samples before calculating Bray–Curtis similarities, resulting in zero‐adjusted Bray–Curtis similarities. Significant factors were further explored using pair‐wise comparisons.

Only individuals containing gastrointestinal contents (food items and/or anthropogenic particles) were included in the PERMANOVA analyses (*n* = 107). Specimens with completely empty stomachs and intestines (*n* = 7) were excluded because no information on diet composition or anthropogenic particle composition was available. Individuals without anthropogenic particles were not excluded, provided that gastrointestinal contents were present.

This analytical approach allowed us to test specific hypotheses regarding diet and contamination drivers. If prey composition and particle ingestion are influenced by spatial habitat use, significant effects of capture zone would be expected. If changes in diet and contamination are driven by cumulative exposure over time, positive relationships with body size would be expected. Likewise, if physiological or behavioral differences between sexes affect diet, ingestion, or particle retention, sex‐related effects would be anticipated.

Conversely, the absence of these effects would suggest that diet remains relatively constant across individuals and that contamination is sufficiently widespread and homogeneous such that biological and ecological traits do not strongly influence exposure.

The homogeneity of multivariate dispersions among capture zones was examined with a PERMDISP test applied to both the diet and the anthropogenic particle Bray–Curtis matrices, using distances to group centroids and 9999 permutations [20]. PERMDISP compares the average distance of the observations to their group centroid and therefore indicates whether the group differences detected by PERMANOVA arise from shifts in location, differences in within‐group dispersion, or both; this distinction is relevant because PERMANOVA can be sensitive to heterogeneous dispersion, particularly in unbalanced designs [35]. Specimens with empty digestive tracts were excluded. All multivariate analyses were conducted in PRIMER v7 with the PERMANOVA + add‐on.

We applied principal coordinates analysis (PCO) to the Bray–Curtis matrix based on diet composition to visualize multivariate patterns among the significant factors. Particle types and prey groups were subsequently overlaid as vectors to examine their association with diet structure. Vector length and direction were used to assess the strength and orientation of these relationships with the ordination axes. For clarity, vectors showing weak relationships with the ordination and clustering near the origin were omitted from the final figure, including anthropogenic particle vectors and less informative prey groups.

## 3. Results

### 3.1. Size Structure

We analyzed 114 individuals of starry round ray, which varied in DW from 9.1 to 25.8 cm, encompassing a wide size range. Kernel density analysis identified three size cohorts: small (< 16.2 cm), medium (16.3–20.2 cm), and large (> 20.3 cm; Figure 2).

**FIGURE 2:**
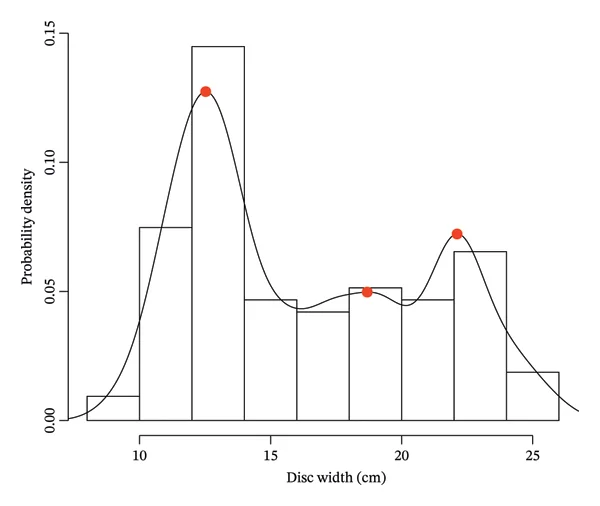
The three distinct cohorts into which the sizes were divided were determined using kernel density.

### 3.2. Diet Composition

We examined 114 stomachs. Seventy‐nine stomachs contained identifiable prey items, and 35 (30.7%) were empty. The diet of the starry round ray consisted primarily of crustaceans (56% FO), followed by cephalopods (11% FO), polychaetes (9% FO), fish (4% FO), and bivalves (2% FO). Crustaceans represented the dominant prey group, occurring in more than half of the stomachs analyzed. Overall, the diet was largely composed of benthic invertebrates.

The PERMANOVA of prey composition indicated that the capture zone was the only factor showing significant differences in diet composition (sex: pseudo‐*F*_1,106_ = 0.45939 0.57868, *p* > 0.05; size: pseudo‐*F*_2,106_ = 1.0025, *p* > 0.05; zone: pseudo‐*F*_2,106_ = 3.888, *p* > 0.05), According to pairwise PERMANOVA comparisons, significant differences were found in prey composition among all three capture zones: southern and central (*t* = 2.0467, *p* > 0.05), northern and central (*t* = 1.9921, *p* > 0.05), and northern versus southern (*t* = 2.5999, *p* > 0.05).

Crustaceans dominated the diet in the northern zone (*n* = 66 prey items), where cephalopods were absent. In the central zone, cephalopods began to appear in the diet (*n* = 17), while crustaceans remained abundant (*n* = 57). In contrast, the southern zone showed a marked increase in cephalopods (*n* = 43) and polychaetes (*n* = 21), accompanied by a decrease in crustaceans (*n* = 14). This shift in prey composition across zones likely underlies the significant differences detected by PERMANOVA.

The PERMDISP test indicated that multivariate dispersion in diet composition also differed among capture zones (*F*_2,104_ = 15.642, P (perm) = 0.0001). The average distance to the group centroid increased from the northern (11.25) through the central (20.3) to the southern zone (28.05), and pairwise comparisons showed significant differences in dispersion between all three zones (northern–central, *p* = 0.0038; northern–southern, *p* = 0.0001; central–southern, *p* = 0.0003), indicating progressively greater multivariate dietary dispersion from north to south.

### 3.3. Anthropogenic Particles

Anthropogenic particles were found in 94 individuals (82% of examined rays), totaling 268 particles for a mean of 2.35 ± 2.21 particles per individual examined (*n* = 114). Among the 94 individuals in which particles were detected, the mean was 2.85 ± 2.11 particles per positive individual. Particle lengths ranged from 0.141 to 4.416 mm. The predominant colors of the particles were blue (59%) and black (28%), followed by red (9%), green (3%), and yellow (1%; Figure 3). Of these particles, 265 (98.9%) were fibers. Blank controls contained only a negligible number of particles (*n* = 6 across all blanks).

**FIGURE 3:**
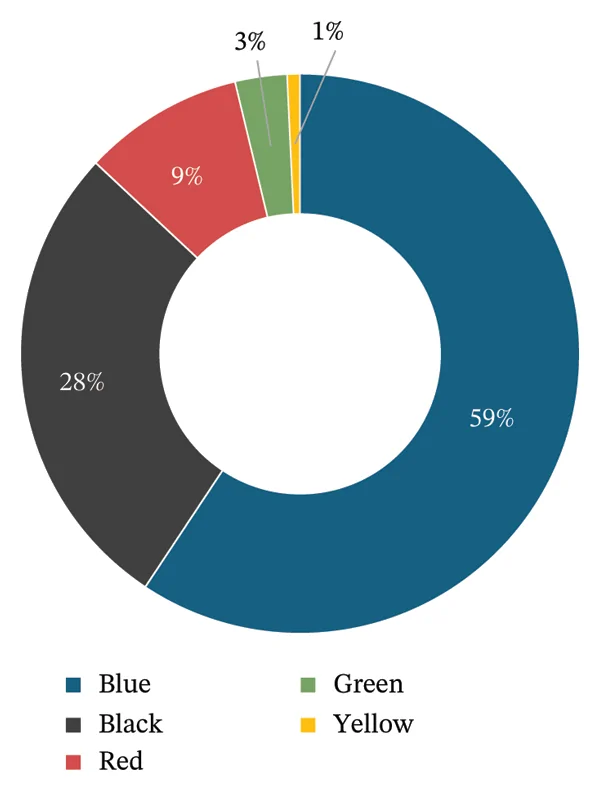
Classification of the different colors of anthropogenic particles observed, represented in percentages.

### 3.4. Differences in Particle Abundances

Particle abundance was best described by the NB GLM, which had the lowest AIC (463.85) and BIC (485.74) of the four models evaluated (Supporting Table S3). The Poisson model fitted poorly because of overdispersion (likelihood‐ratio test against the NB: *χ* ^2^ = 27.3, *p* = 0.0001), whereas the zero‐inflated NB did not improve on the NB (equal log‐likelihood; ΔAIC = + 2.0), indicating that the individuals without particles were consistent with the NB count process rather than forming a separate class of structural zeros. The hurdle model achieved a marginally higher log‐likelihood but a higher AIC (471.79) owing to its additional parameters. Inference was therefore based on the NB model fitted to all 114 individuals.

Particle abundance did not differ significantly among size classes (LR *χ* ^2^(2) = 1.15, *p* = 0.562), between sexes (LR *χ* ^2^(1) = 0.15, *p* = 0.701), among sampling zones (LR *χ* ^2^(2) = 2.69, *p* = 0.261), or with dietary diversity (LR *χ* ^2^(1) = 1.60, *p* = 0.206). Estimated marginal means were similar across all factor levels (size classes, 1.9–2.6 particles; sexes, 2.3–2.5; zones, 2.0–3.0; Supporting Table S3), and none of the differences were significant. Modeling DW as a continuous predictor gave the same result (NB: *β* = −0.027, *p* = 0.297), in agreement with the Spearman correlation (*ρ* = −0.092, *p* = 0.332); we found no evidence that particle abundance increased with body size. Overall, anthropogenic particle abundance in the digestive tract did not vary significantly with size, sex, sampling zone, or diet.

### 3.5. Chemical Characteristics of Particles

µFTIR–ATR analysis classified the 268 anthropogenic particles found in the digestive tracts of *U. asterias* into three groups according to their origin (Table 1). Natural fibers were the most abundant, accounting for 84.33% of all particles, and consisted of cellulose (46.64%) and cotton (37.69%). Semisynthetic fibers, represented entirely by rayon (a regenerated cellulose fiber), made up 2.24%. Synthetic polymers accounted for the remaining 13.43%; within this group, polyester (10.07%) was the most abundant, followed by polypropylene (1.12%), acrylic (1.12%), poly(ethyl acrylate–acrylamide) (0.37%), polyethylene–propylene (0.37%), and polyester urethane (0.37%).

**TABLE 1:** Number of types of anthropogenic particles found in the digestive tracts of female and male *U. asterias*.

	Cellulose	Cotton	Polyester	Rayon	Polypropylene	Acrylic	PAA	PEP	Polyester urethane	Total
Female	62	44	14	5	2	2	0	0	0	129
Male	63	57	13	1	1	1	1	1	1	139
Total	125	101	27	6	3	3	1	1	1	268

*Note:* PAA, poly (ethyl acrylate–acrylamide); PEP, polyethylene–propylene.

In contrast to our initial hypothesis, the PERMANOVA indicated no influence of sex, size, or capture zone on anthropogenic particle composition (*n* = 107 individuals; sex: pseudo‐*F*_1,106_ = 0.74024, *p* > 0.05; size: pseudo‐*F*_2,106_ = 0.97923, *p* > 0.05; zone: pseudo‐*F*_2,106_ = 0.3313, *p* > 0.05).

For anthropogenic particle composition, the PERMDISP test showed significant differences in multivariate dispersion among capture zones (*F*_2,106_ = 6.08, P (perm) = 0.0022). Pairwise PERMDISP comparisons showed significant differences between the central and northern zones (*p* = 0.0007) and between the central and southern zones (*p* = 0.0289), whereas the northern and southern zones did not differ significantly (*p* = 0.2161). Thus, although within‐zone variability differed spatially, PERMANOVA provided no evidence of significant differences in particle composition among capture zones.

### 3.6. Spatial Patterns in Diet Composition

The PERMANOVA of prey composition indicated that capture zone was the only factor showing significant differences in diet composition, whereas sex and size did not show significant effects. Therefore, a PCO based on prey composition was performed to further explore spatial differences in diet among capture zones. Initially, prey groups and anthropogenic particle types were fitted as vectors onto the ordination. However, some prey groups and anthropogenic particle types showed weak relationships with the ordination axes, with their vectors clustering near the origin. Accordingly, these vectors were omitted from Figure 4 for clarity.

**FIGURE 4:**
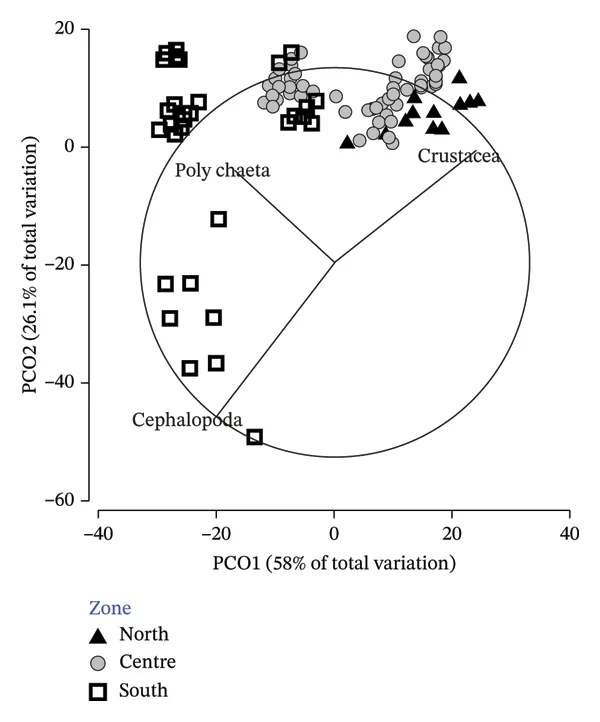
Principal coordinate analysis (PCO) based on prey composition of the starry round ray from different capture zones. Prey groups were fitted as vectors to explore their association with dietary patterns; only vectors showing clear relationships with the ordination axes are displayed.

The PCO (Figure 4) showed partial separation among capture zones, although overlap was also observed. Individuals from the northern zone were mainly distributed in the direction of the crustacean vector, consistent with the predominance of this prey group in their diet. Individuals from the central zone occupied an intermediate region of the ordination and overlapped with samples from both the northern and southern zones. In contrast, individuals from the southern zone showed a broader distribution in the ordination space and were more strongly associated with the cephalopod and polychaete vectors.

## 4. Discussion

This study is the first to examine anthropogenic particles in the genus *Urotrygon*, and its findings contribute to understanding the patterns of anthropogenic particle contamination to which benthic organisms in the Gulf of California may be exposed, complementing previous information reported for elasmobranchs in this region [7, 11] (Supporting Table S4).

### 4.1. Diet Composition and Anthropogenic Particles

The differences observed among capture zones suggest spatial variation in prey use along the Sinaloa coastline, which may reflect differences in prey availability and benthic community structure. Similar regional variation in diet composition has been reported between northern and southern populations in the Mexican Pacific [36]. In addition, cephalopods are described here for the first time as part of the diet of the starry round ray, suggesting a broader trophic niche than previously recognized.

Despite this, crustaceans remain the dominant prey, consistent with previous reports [36] and with the general characterization of Urotrygonidae as crustacean predators [37]. However, these prey organisms have been shown to contain anthropogenic particles in different tissues [38]; therefore, trophic transfer through prey consumption may represent an indirect pathway of anthropogenic particle intake in the starry round ray.

Dietary dispersion increased progressively from the northern to the southern zone, suggesting greater among‐individual dietary variability toward the south. This pattern is consistent with the prey data, as diets in the northern zone were strongly dominated by crustaceans, whereas the southern zone showed a broader representation of prey groups, particularly cephalopods and polychaetes. Thus, the spatial differences detected by PERMANOVA likely reflect both differences in multivariate diet structure and differences in within‐zone dietary variability.

We found particles in 82% of the starry round rays from coastal waters off Sinaloa, in the southeastern Gulf of California, with an average of 2.35 particles per individual. This is one of the highest rates reported for batoid species anywhere and is much higher than the 32.4% reported for *Urobatis halleri* in the same region [7]. It is also similar to the 79.3% found in juvenile *Sphyrna lewini* from the coasts of Sinaloa [21]. Together, these results show that particle contamination is consistent across different elasmobranch species, feeding habits, and trophic levels along this coastal region. The enormous difference between *U. asterias* (82%) and *U. halleri* (32.4%) should be interpreted carefully. It likely reflects a mix of methodological, ecological, spatial, and temporal factors rather than a single cause. One key difference is that we included natural fibers in our analysis. In our samples, 84.33% of the particles were natural fibers, namely cellulose and cotton, 2.24% were semisynthetic rayon, and only 13.43% were synthetic polymers. If natural fibers were undercounted or excluded in the *U. halleri* study, that alone could explain much of the difference in occurrence rates. The two species also differ in substrate use, which could partly explain the difference in occurrence. *U. halleri* is mostly restricted to sandy bottoms in shallow coastal waters, whereas the starry round ray also uses muddy substrates. This broader substrate use by the starry round ray could result in additional exposure pathways not shared by *U. halleri*. The studies also covered different parts of the region and different seasons. The baseline study focused on the north–central coast of Sinaloa (the Santa María La Reforma estuarine system), while ours included the northern, central, and southern part of that coast. They were also conducted in different years and at different times of the year. Fishing activity, tourism, wastewater discharge, and other human activities vary across space and season, and these could influence contamination levels. Without sampling the same places at the same times, it is not possible to separate these effects clearly.

### 4.2. µFTIR–ATR Analysis

Our use of µFTIR–ATR spectroscopy to confirm particle composition represents an important methodological technique for batoid contamination studies. While visual identification of particles is common in anthropogenic particle research, spectroscopic confirmation is essential for distinguishing natural from synthetic fibers and assigning specific material types, distinctions that are critical for source attribution and management planning.

One of the most notable findings of our study is the dominance of natural fibers. Cellulose (Figure 5a) and cotton (Figure 5b) together accounted for more than 84% of all particles. This pattern is similar to that reported by Malthaner et al. [21] in juvenile *S*. *lewini* from the Santa María La Reforma estuarine system. These results suggest that this coastal area of Sinaloa has a strong signal of fiber contamination, especially from textiles.

**FIGURE 5:**
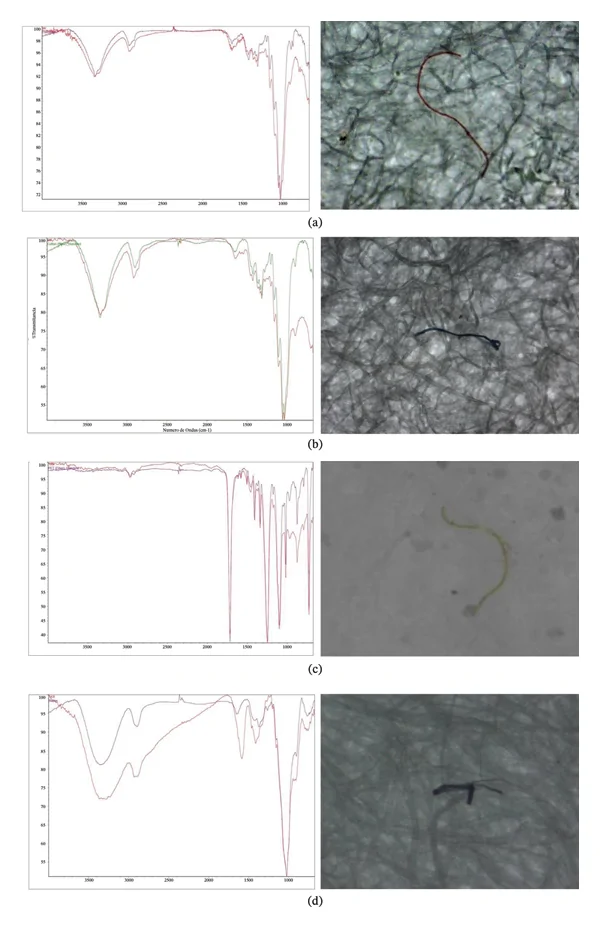
Representative µFTIR–ATR spectra of the main anthropogenic particle types recovered from the digestive tract of *Urotrygon asterias*: (a) cellulose, (b) cotton, (c) polyester, and (d) rayon. Cellulose and cotton were the dominant materials (together > 84% of all particles).

This challenges the common focus on synthetic plastics alone. Natural fibers are often assumed to be harmless; however, they are also linked to human activity and can persist in the environment. The three groups differ in environmental fate: synthetic polymers such as polyester are the most persistent and degrade only slowly, semisynthetic rayon occupies an intermediate position, and natural fibers such as cellulose and cotton are generally the most biodegradable. This gradient, however, does not make natural fibers innocuous because they undergo industrial processing during which they are frequently treated with pigments and chemical additives [39]. Such substances can leach into biological tissues and may cause adverse effects in marine organisms, indicating that their presence should not be dismissed as harmless despite their nonsynthetic origin.

Among the synthetic polymers, polyester was the dominant material (10.07% of total particles; Figure 5c). This is consistent with global patterns, as polyester is the most widely produced synthetic fiber worldwide and is extensively used in textiles [40]. Rayon, in contrast, is a semisynthetic fiber: It is manufactured from regenerated cellulose and therefore occupies an intermediate position between fully synthetic polymers and natural fibers in terms of composition and environmental behavior. Although it represented a small fraction of the particles recovered (2.24%; Figure 5d), it has already been reported in both demersal and pelagic fish [41]. The relatively low proportion of synthetic polymers compared with natural fibers may reflect the actual composition of environmental contamination; therefore, excluding natural fibers could lead to an underestimation of anthropogenic particle contamination.

### 4.3. Particles Sources

Because this study did not include direct environmental measurements or source‐tracking analyses, the exact sources of the anthropogenic particles found in the digestive tract of *U. asterias* could not be determined with certainty. However, given the composition of the particles identified in this study, and considering the relevance of certain anthropogenic activities in the study area, it is possible that these activities contribute to the particle contamination observed in the region.

The likely sources of these fibers include domestic laundry, wastewater discharge, fishing activities, and tourism. Cellulose‐based fibers such as rayon and cotton are released during washing and can pass through wastewater treatment systems into coastal waters. In Sinaloa, effluents generated by key economic activities are commonly discharged into coastal ecosystems [42]. This has already been documented in the Urías estuarine system, southern Sinaloa, where increased particle abundance at certain sites has been linked to wastewater discharges [43].

Fishing activities may also represent an important source of fiber. Crew clothing can release textile fibers, while several components of fishing gear, including nets, lines, floats, and buoys, are manufactured from polymers and are frequently lost or discarded at sea [44, 45]. Given that fishing in the Gulf of California is a key economic activity, this pathway is particularly relevant, as the state of Sinaloa ranks second nationally in total fish production, accounting for 20.52% of the national volume [46].

Tourism may further intensify fiber inputs. During peak summer seasons in Gulf coastal areas, increased laundry use and wastewater discharge can elevate fiber release. Sánchez‐Paz et al. [47], for example, reported higher particle abundances following major holiday periods. This pattern is especially relevant in Mazatlán, one of the most intensively visited coastal destinations in the country, which receives nearly three million visitors annually [48], potentially increasing local fiber loads.

Previous work on *U. halleri* [7] showed that sediments in the Santa María La Reforma estuarine system contain notable levels of microplastics, especially fibers. This is important because benthic rays feed directly in sediments, which act as a reservoir of particles. It helps explain why fibers associated with sediments are more common than particles floating in the water column.

### 4.4. Exposure Pathways and Drivers

We also found that contamination did not vary with sex, body size, diet, or capture zone. The absence of sex‐related differences suggests that exposure routes and ingestion pathways are highly similar between males and females. One likely explanation is the similar spatial distribution and feeding behavior reported between sexes in this species in previous studies [36, 49, 50].

Furthermore, particles may not remain long in the digestive tract. Experimental studies in teleost fishes indicate that most microplastics can be excreted within approximately 25 h after ingestion [51]. This may suggest that the contamination observed reflects recent exposure rather than lifetime accumulation, particularly in benthic rays that are continuously exposed to sediment‐associated particles.

In this context, ingestion may occur passively during feeding. The starry round ray excavates sediment to locate invertebrates, a behavior described in other ray species [52], and in doing so likely ingests particles along with sediment. If ingestion is mostly incidental and associated with sediment contact rather than prey type, contamination would likely not depend strongly on diet, as observed in the present study.

Although diet differed across regions, particle loads remained similar. This may indicate that exposure is relatively uniform along the coast of Sinaloa. Accordingly, this consistency across differing diets could suggest widespread contamination of benthic sediments in this region, such that benthic‐feeding rays would likely encounter anthropogenic particles regardless of diet; however, direct sediment sampling would be needed to confirm this pathway.

### 4.5. Limitations and Future Research

A key limitation of our study is that we did not collect sediment or water samples at the same time and places as the animals. Without that environmental context, we cannot tell whether contamination is truly uniform across the region or whether biological processes make exposure appear uniform. These two explanations have different management implications. If contamination is dispersed in the environment, broad regional action is needed. If it is patchy but exposure becomes uniform because of feeding behavior, then protecting or restoring certain habitats might help. Future studies should sample both animals and their environment at the same time to clarify this.

A further limitation concerns covariates that were not recorded during the survey. Individuals were characterized by sex and DW, but maturity stage was not determined; because reproductive stage can influence feeding rate, movement, and metabolic demand independently of body size, unmeasured variation in maturity may obscure size‐ or sex‐related patterns in particle ingestion. Likewise, capture depth and the specific microhabitat or substrate type at each site were not registered, even though these factors govern the local availability of sediment‐associated fibers and could therefore produce individual differences in exposure that our design cannot resolve. Together with the absence of paired sediment sampling noted above, these unmeasured factors constrain the ecological interpretation of the biological and spatial patterns, and future surveys should record maturity stage, capture depth, and habitat/substrate characteristics so that their effects can be separated from those examined here.

Besides the former, the short sampling window is also a limitation that needs to be acknowledged. Our data comes from July and August of a single year. Contamination levels may change with seasons. Summer in the Gulf often brings high tourist activity, which may increase fiber inputs. Other seasons, such as peak fishing periods, may shift the types and amounts of particles entering the system. Because both our study and earlier work on *U*. *halleri* were based on single seasons, we still lack a clear picture of how contamination changes over time. Multiseason and multiyear monitoring is needed to understand whether these high levels are stable, increasing, or seasonal peaks.

Additionally, dietary diversity was low across the sampled population, with 88.6% of individuals showing a Shannon index of zero, reflecting either empty stomachs or diets dominated entirely by a single prey type. This limited variability likely reduced statistical power to detect a diet‐particle relationship. Pooling data across multiple sampling seasons or sites could help capture a broader range of dietary conditions, improving the ability to detect diet‐related patterns.

Sample sizes were unbalanced across factor levels, particularly for sampling zone (65 individuals in the central zone vs. 33 in the south and 16 in the north) and size class (62 small, 30 large, and 22 medium individuals). Under these conditions, nonsignificant results should be interpreted with caution, as less‐represented levels do not provide sufficient observations to precisely estimate group‐level variability. Future studies comparing anthropogenic particle concentrations across biological and spatial factors should aim for balanced representation across factor levels, ideally informed by a priori power analysis to estimate the required sample size per category.

Because PERMANOVA is sensitive to heterogeneous dispersions in unbalanced designs [35], the significant zone effect on diet composition should be interpreted as a combined difference in average composition and within‐zone variability rather than as a difference in average composition alone. In contrast, PERMANOVA detected no significant zone effect on anthropogenic particle composition despite significant heterogeneity in dispersion; therefore, this result does not alter the overall conclusion regarding the absence of significant spatial differences in particle composition.

### 4.6. Ecological Risk Considerations

From a conservation perspective, the occurrence of anthropogenic particles observed in the starry round ray raises potential implications that require further evaluation. Particle occurrence was not restricted to a particular demographic group within the sampled population, as particles were detected in individuals of different sizes and in both sexes. In addition, this species is frequently caught as bycatch in shrimp trawl fisheries in the Gulf of California [53], and individuals may therefore be simultaneously subjected to fishing pressure and exposure to anthropogenic particles. Although the observed occurrence may be consistent with recurrent exposure, sampling during a single season does not allow us to determine whether such exposure is persistent. Therefore, future studies should evaluate whether these stressors may interact and generate potential biological or population‐level effects through multitemporal sampling, more balanced designs, and direct assessments of their biological consequences.

Fibers are among the most frequently reported anthropogenic particles in aquatic ecosystems [54]. Although biological effects were not evaluated in the present study, available experimental evidence suggests that exposure to fibers can produce variable biological responses among species. For example, Mizukami‐Murata et al. [55] reported that a 21‐day exposure to fibers did not cause mortality in *Oryzias latipes*; however, it resulted in reductions in body length and weight. They also demonstrated that fiber size influenced retention within the gastrointestinal tract, with longer fibers showing a greater tendency to remain in the digestive system. Similarly, Hu et al. [56] detected no changes in growth or in the gonadosomatic and hepatosomatic indices of exposed fish, yet histological examination revealed marked gill alterations, including epithelial detachment from the gill arches, fusion of primary lamellae, increased mucus production, aneurysms in the secondary lamellae, epithelial lifting, and swelling of the opercular membrane. These findings indicate that the absence of visible effects at the organism level does not necessarily imply the absence of biological damage, as adverse effects may occur at the tissue or cellular level.

The magnitude of these effects also appears to depend on fiber type and their physical and chemical characteristics. MacAulay et al. [14] reported higher mortality in fish exposed to synthetic polyester fibers than in fish exposed to natural cotton and bamboo fibers. They also documented changes in host–parasite interactions and suggested that part of these effects could be associated with dyes incorporated into the fibers. In contrast, Bunge et al. [13] found no alterations in growth or immune parameters in *Gasterosteus aculeatus* exposed to high concentrations of virgin polyester and cotton fibers and reported efficient fiber egestion through feces. Collectively, these studies indicate that the biological effects associated with fiber exposure are not uniform and are likely influenced by multiple factors, including the study species, exposure conditions, the physical characteristics of the fibers, and the presence of chemical additives.

Although several studies have documented the ingestion of anthropogenic particles in elasmobranchs, evidence regarding their biological consequences remains very limited. Most of these investigations have focused on documenting the occurrence, abundance, and characterization of ingested particles. Consequently, the level of risk posed by this contamination to sharks and rays remains unknown [6]. Therefore, future research should move beyond occurrence studies and evaluate the physiological, histopathological, and ecological consequences of anthropogenic particle exposure in elasmobranchs.

### 4.7. Management Implications

From a management perspective, the lack of differences among regions or demographic groups suggests that targeted measures alone will not be enough. Reducing inputs at their source is essential. This includes improving wastewater treatment to capture microfibers, reducing loss of fishing gear, strengthening waste management in coastal communities, and regulating wastewater from tourist facilities. Some governments, such as the European Union, are already promoting reforms aimed at reducing the negative impact of these particles on ecosystems, including bans on single‐use plastics, increased use of recyclable packaging, and improvements in waste collection systems for these materials [57].

Furthermore, long‐term monitoring programs that include multiple species, seasons, and environmental samples are also needed. Because contamination appears to affect several elasmobranch species, including *U. asterias*, *U*. *halleri*, and *S*. *lewini*, the issue should be addressed at the ecosystem level rather than focusing on a single species.

Overall, our study shows that anthropogenic particle contamination in the starry round ray collected from three coastal regions of Sinaloa and the southeastern Gulf of California is common and dominated by natural fibers. The absence of significant differences across sex, size, diet, and sampling regions suggests similar levels of exposure to anthropogenic particles among the individuals evaluated during the study period. However, paired environmental sampling and broader temporal coverage are needed to better understand the mechanisms underlying these patterns. Together with findings from other elasmobranch species from the coast of Sinaloa, our results suggest that exposure to anthropogenic particles may be a shared phenomenon among different species from this coastal region. Addressing it will require coordinated regional action, long‐term monitoring, and deeper research into the biological consequences of both synthetic and natural fibers.

## 5. Conclusions

This study provides the first assessment of anthropogenic particle contamination in the genus *Urotrygon*, documenting a very high occurrence (82%) and a predominance of natural fibers (84%) in *U. asterias* specimens collected from the coastal waters of Sinaloa, in the southern Gulf of California. Consistent with the patterns discussed above, no significant effects of sex, size, sampling zone, or dietary diversity were detected, suggesting broadly similar exposure levels among the individuals evaluated; however, the lack of paired environmental data limits the interpretation of the mechanisms underlying these patterns. Comparison with the baseline study on *U. halleri* [7] suggests that the batoid species evaluated to date along the coast of Sinaloa may be exposed to anthropogenic particles. Differences in occurrence rates and material composition among studies may be influenced by methodological, ecological, spatial, and temporal factors. The temporal limitation of sampling during a single season in both studies highlights the need for multiseasonal and multiyear monitoring to characterize temporal variation and long‐term trends. From a conservation perspective, exposure to anthropogenic particles was observed across all demographic groups evaluated, highlighting the importance of implementing strategies to reduce contamination sources and to assess their potential biological effects. Together with recent findings in *S. lewini* [21], our results provide additional evidence that anthropogenic particle contamination represents a relevant concern for the coastal ecosystems of Sinaloa and warrants attention in terms of management and long‐term monitoring. Future research should prioritize paired organism–environment sampling, temporal replication, interspecific comparisons, and the assessment of biological effects to improve our understanding of contamination dynamics and their ecological consequences in this marine ecosystem.

## Author Contributions

Allan Rosales‐Valencia: conceptualization, data curation, formal analysis, investigation, methodology, validation, visualization, and writing–original draft. Felipe Amezcua: conceptualization, data curation, formal analysis, funding acquisition, investigation, methodology, visualization, and writing–review and editing. Carlos René Green‐Ruíz: investigation, funding acquisition, and methodology.

## Funding

This study was supported by the Universidad Nacional Autónoma de México funded this research through the Instituto de Ciencias del Mar y Limnología and the institutional grants 2024 and 2025 awarded to Felipe Amezcua and Carlos Green.

## Disclosure

All authors approved the final version of the manuscript to be published.

## Conflicts of Interest

The authors declare no conflicts of interest.

## Supporting Information

Additional supporting information can be found online in the Supporting Information section.

## Supporting information


**Supporting Information** Supporting 1. ST1. Anthropogenic particles detected in procedural blank controls during sample processing, including material identification and particle size. Supporting 2. ST2. Recovery and µFTIR–ATR identification results for fibers used as positive controls, including fiber color and spectral match percentage. Supporting 3. ST3. Comparison of candidate count models and results of the negative binomial generalized linear model (GLM) for anthropogenic particle abundance in *Urotrygon asterias*, including results for biological and spatial predictors. Supporting 4. ST4. Comparison of anthropogenic particle contamination reported in elasmobranch studies from the Gulf of California, including occurrence, material composition, sampling periods, and limitations.

## Data Availability

The data that support the findings of this study are available from the corresponding author upon reasonable request.

## References

[1] Plastics Europe , Plastics–The Fast Facts 2024, 2024, https://plasticseurope.org/knowledge-hub/plastics-the-fast-facts-2024/.

[2] Andrady A. L. , Microplastics in the Marine Environment, Marine Pollution Bulletin. (2011) 62, no. 8, 1596–1605, 10.1016/j.marpolbul.2011.05.030. 21742351

[3] Andrady A. L. , The Plastic in Microplastics: A Review, Marine Pollution Bulletin. (2017) 119, no. 1, 12–22, 10.1016/j.marpolbul.2017.01.082. 28449819

[4] Kakade A. , Mi J. , and Long R. , Microplastics in the World Oceans and Strategies for Their Control, Reviews of Environmental Contamination & Toxicology. (2024) 262, no. 1, 10.1007/s44169-024-00069-x.

[5] Alomar C. , Estarellas F. , and Deudero S. , Microplastics in the Mediterranean Sea: Deposition in Coastal Shallow Sediments, Spatial Variation and Preferential Grain Size, Marine Environmental Research. (2016) 115, 1–10, 10.1016/j.marenvres.2016.01.005. 26803229

[6] Parton K. J. , Godley B. J. , Santillo D. , Tausif M. , Omeyer L. C. M. , and Galloway T. S. , Investigating the Presence of Microplastics in Demersal Sharks of the North-East Atlantic, Scientific Reports. (2020) 10, no. 1, 10.1038/s41598-020-68680-1. PMC7376218 32699221

[7] Pinho I. , Amezcua F. , Rivera J. M. , Green-Ruiz C. , De Jesus Piñón-Colin T. , and Wakida F. , First Report of Plastic Contamination in Batoids: Plastic Ingestion by Haller’s Round Ray (*Urobatis halleri*) in the Gulf of California, Environmental Research. (2022) 211, 10.1016/j.envres.2022.113077. 35276199

[8] Stefanelli-Silva G. , Friedemann P. , De Moraes B. R. et al., Bottom-Feeders Eat Their Fiber: Ingestion of Anthropogenic Microdebris by Antarctic Deep-Sea Invertebrates Depends on Feeding Ecology, Environmental Science and Technology. (2024) 58, no. 50, 22355–22367, 10.1021/acs.est.4c09487. PMC11656696 39568238

[9] Frias J. and Nash R. , Microplastics: Finding a Consensus on the Definition, Marine Pollution Bulletin. (2019) 138, 145–147, 10.1016/j.marpolbul.2018.11.022. 30660255

[10] Santini S. , De Beni E. , Martellini T. et al., Occurrence of Natural and Synthetic Micro-Fibers in the Mediterranean Sea: A Review, Toxics. (2022) 10, no. 7, 10.3390/toxics10070391. PMC9320265 35878296

[11] Collard F. , Gasperi J. , Gilbert B. et al., Anthropogenic Particles in the Stomach Contents and Liver of the Freshwater Fish Squalius cephalus, The Science of the Total Environment. (2018) 643, 1257–1264, 10.1016/j.scitotenv.2018.06.313. 30189542

[12] Athey S. N. and Erdle L. M. , Are We Underestimating Anthropogenic Microfiber Pollution? A Critical Review of Occurrence, Methods, and Reporting, Environmental Toxicology and Chemistry. (2022) 41, no. 4, 822–837, 10.1002/etc.5173. 34289522

[13] Bunge A. , Lugert V. , McClure M. , Kammann U. , Hanel R. , and Scharsack J. P. , Less Impact Than Suspected: Dietary Exposure of Three-Spined Sticklebacks to Microplastic Fibers Does not Affect Their Body Condition and Immune Parameters, The Science of the Total Environment. (2022) 819, 10.1016/j.scitotenv.2022.153077. 35038536

[14] MacAulay S. , Masud N. , Davies-Jones J. , Ward B. D. , and Cable J. , The Impacts of Synthetic and Cellulose-Based Fibres and Their Associated Dyes on Fish Hosts and Parasite Health, Environmental Science and Pollution Research. (2023) 30, no. 58, 121558–121568, 10.1007/s11356-023-30794-0. PMC10724321 37955728

[15] Détrée C. , Labbé C. , Paul-Pont I. et al., On the Horns of a Dilemma: Evaluation of Synthetic and Natural Textile Microfibre Effects on the Physiology of the Pacific Oyster *Crassostrea gigas* , Environmental Pollution. (2023) 331, no. Pt 2, 10.1016/j.envpol.2023.121861. 37245792

[16] Barrick A. , Boardwine A. J. , Nguyen N. H. , Sevcu A. , Novotna J. , and Hoang T. C. , Acute Toxicity of Natural and Synthetic Clothing Fibers Towards Daphnia Magna: Influence of Fiber Type and Morphology, The Science of the Total Environment. (2025) 967, 10.1016/j.scitotenv.2025.178751. 39954472

[17] Hermsen E. , Mintenig S. M. , Besseling E. , and Koelmans A. A. , Quality Criteria for the Analysis of Microplastic in Biota Samples: A Critical Review, Environmental Science and Technology. (2018) 52, no. 18, 10230–10240, 10.1021/acs.est.8b01611. PMC6146318 30137965

[18] Kosuth M. , Simmerman C. B. , and Simcik M. , Quality Assurance and Quality Control in Microplastics Processing and Enumeration, Environmental Engineering Science. (2023) 40, no. 11, 605–613, 10.1089/ees.2023.0063.

[19] Cruz-García R. and Amezcua F. , A Standardized Protocol for Microplastic and Non-Synthetic Microfiber Extraction and µFTIR Identification in Elasmobranch Gastrointestinal Tissues, MethodsX. (2026) 16, 10.1016/j.mex.2026.103996. PMC13264173 42292864

[20] Munno K. , Hoopes L. , Lyons K. , Drymon M. , Frazier B. , and Rochman C. M. , High Microplastic and Anthropogenic Particle Contamination in the Gastrointestinal Tracts of Tiger Sharks (*Galeocerdo cuvier*) Caught in the Western North Atlantic Ocean, Environmental Pollution. (2024) 344, 10.1016/j.envpol.2023.123185. 38147950

[21] Malthaner L. , Garcia X. , Rios-Mendoza L. M. , Rivera-Hernández J. R. , Cruz R. , and Amezcua F. , First Data on Anthropogenic Microparticles in the Gastrointestinal Tract of Juvenile Scalloped Hammerhead Sharks (*Sphyrna lewini*) in the Gulf of California, Fishes. (2024) 9, no. 8, 10.3390/fishes9080310.

[22] Pegado T. , Brabo L. , Schmid K. et al., Ingestion of Microplastics by *Hypanus guttatus* Stingrays in the Western Atlantic Ocean (Brazilian Amazon Coast), Marine Pollution Bulletin. (2021) 162, 10.1016/j.marpolbul.2020.111799. 33183749

[23] De Pinho J. V. , Melo A. , Scarpa J. et al., First Report on Microplastics Ingestion in the Critically Endangered Spiny Butterfly Ray *Gymnura altavela* (Elasmobranchii, Gymnuridae) from a Southwestern Atlantic Estuary, Environmental Pollution. (2026) 392, 10.1016/j.envpol.2025.127564. 41421471

[24] Lu H. , Smith J. L. , Ziajahromi S. , and Leusch F. D. , Microplastics and Other Anthropogenic Fibres in Large Apex Shark Species: Abundance, Characteristics, and Recommendations for Future Research, Chemosphere. (2024) 349, 10.1016/j.chemosphere.2023.140957. 38128742

[25] Ehemann N. R. , Hernández S. , Siccha‐Ramírez R. , García‐Rodríguez F. J. , and De la Cruz‐Agüero J. , Alike but Genetically Divergent: The Resurrection of Urotrygon asterias (Jordan & Gilbert, 1883) From Its Closest Relatives, the Munda and the Blotched Stingray, Journal of Fish Biology. (2024) 105, no. 4, 1044–1059, 10.1111/jfb.15864. 38978288

[26] Alvarado-Marín A. , Saavedra-Sotelo N. C. , and Amezcua F. , Mapping Marine Identities: Integrating Morphometric and Molecular Data to Distinguish Similar Round Ray Species (Urotrygonidae) in the Southeastern Gulf of California, Regional Studies in Marine Science. (2026) 101, 10.1016/j.rsma.2026.105247.

[27] Munno K. , Lusher A. L. , Minor E. C. et al., Patterns of Microparticles in Blank Samples: A Study to Inform Best Practices for Microplastic Analysis, Chemosphere. (2023) 333, 10.1016/j.chemosphere.2023.138883. PMC11181520 37169088

[28] Lam T. W. L. , Ho H. T. , Th A. M. A. , and Fok L. , Microplastic Contamination of Surface Water-Sourced Tap Water in Hong Kong—A Preliminary Study, Applied Sciences. (2020) 10, no. 10, 10.3390/app10103463.

[29] Lordachescu L. , Papacharalampos K. , Barritaud L. et al., Microplastics in an Advanced Wastewater Treatment Plant: Sustained and Robust Removal Rates Unfazed by Seasonal Variations, Microplastics And Nanoplastics. (2024) 4, no. 1, 10.1186/s43591-024-00097-3.

[30] Jafarova M. , Aherne J. , and Loppi S. , Regional Sources Drive Atmospheric Microplastic Deposition at Rural Background Sites, Environmental Research. (2025) 285, no. Pt 5, 10.1016/j.envres.2025.122663. 40846197

[31] Silverman B. W. , Density Estimation for Statistics and Data Analysis, 1986, CRC Press.

[32] Hyslop E. J. , Stomach Contents Analysis a Review of Methods and Their Application, Journal of Fish Biology. (1980) 17, no. 4, 411–429, 10.1111/j.1095-8649.1980.tb02775.x.

[33] Venables W. N. and Ripley B. D. , Modern Applied Statistics with S, 2002, 4th edition, Springer, New York, NK, 10.1007/978-0-387-21706-2.

[34] Zeileis A. , Kleiber C. , and Jackman S. , Regression Models for Count Data in R, Journal of Statistical Software. (2008) 27, no. 8, 1–25, 10.18637/jss.v027.i08.

[35] Anderson M. J. and Walsh D. C. I. , PERMANOVA, ANOSIM, and the Mantel Test in the Face of Heterogeneous Dispersions: What Null Hypothesis are You Testing?, Ecological Monographs. (2013) 83, no. 4, 557–574, 10.1890/12-2010.1.

[36] Oñate-González E. , Amezcua F. , Buszkiewicz J. , Castellanos-Cendales A. , and Amezcua-Linares F. , Trophic Ecology of the Blotched Stingray, *Urotrygon chilensis* (Elasmobranchii: Myliobatiformes: Urotrygonidae), in Three Areas of the Mexican Pacific, Acta Ichthyologica et Piscatoria. (2017) 47, no. 2, 185–196, 10.3750/aiep/02099.

[37] Silva-Garay L. , Pacheco A. S. , and Vélez-Zuazo X. , First Assessment of the Diet Composition and Trophic Level of an Assemblage of Poorly Known Chondrichthyans off the Central Coast of Peru, Environmental Biology of Fishes. (2018) 101, no. 10, 1525–1536, 10.1007/s10641-018-0797-0.

[38] Valencia-Castañeda G. , Ibáñez-Aguirre K. , Rebolledo U. A. , Capparelli M. V. , and Páez-Osuna F. , Microplastic Contamination in Wild Shrimp *Litopenaeus Vannamei* From the Huizache-Caimanero Coastal Lagoon, SE Gulf of California, Bulletin of Environmental Contamination and Toxicology. (2022) 109, no. 3, 425–430, 10.1007/s00128-022-03568-6. 35786731

[39] Mather R. R. and Wardman R. H. , The Chemistry of Textile Fibres, 2015, Royal Society of Chemistry Books Gateway, 10.1039/9781782626534.

[40] Smelik A. , Polyester: A Cultural History, Fashion Practice. (2023) 1–21, no. 2, 279–299, 10.1080/17569370.2023.2196158.

[41] Lusher A. , McHugh M. , and Thompson R. , Occurrence of Microplastics in the Gastrointestinal Tract of Pelagic and Demersal Fish From the English Channel, Marine Pollution Bulletin. (2013) 67, no. 1-2, 94–99, 10.1016/j.marpolbul.2012.11.028. 23273934

[42] Leyva-Morales J. B. , De Jesús Bastidas-Bastidas P. , Del Carmen Salvatierra-Stamp V. et al., Temporal Distribution and Ecological Risk Assessment for Pesticides in Water From the North-Central Coastal Zone of Sinaloa, Mexico, Heliyon. (2024) 10, no. 15, 10.1016/j.heliyon.2024.e35207. PMC11337721 39170137

[43] Ruiz-Fernández A. C. , Pérez-Bernal L. H. , Sanchez-Cabeza J. , Valencia-Castañeda G. , Ontiveros-Cuadras J. F. , and Alonso-Hernández C. M. , Accelerating Microplastic Contamination in 210Pb Dated Sediment Cores From an Urbanized Coastal Lagoon (NW Mexico) Since the 1990s, The Science of the Total Environment. (2024) 951, 10.1016/j.scitotenv.2024.175613. 39168330

[44] Li W. , Tse H. , and Fok L. , Plastic Waste in the Marine Environment: A Review of Sources, Occurrence and Effects, The Science of the Total Environment. (2016) 566–567, 333–349, 10.1016/j.scitotenv.2016.05.084. 27232963

[45] Gesamp , Gilardi K. , Sea-Based Sources of Marine Litter, 2021.

[46] Conapesca , Anuario Estadístico de Acuacultura y Pesca 2023, 2023, Mazatlán, Sinaloa, México, https://nube.conapesca.gob.mx/sites/cona/dgppe/2023/ANUARIO_ESTADISTICO_DE_ACUACULTURA_Y_PESCA_2023.pdf.

[47] Sánchez-Paz A. , Encinas-García T. , Ávila-Félix L. , and Mendoza-Cano F. , Microplastics in Surface Waters Surrounding a Touristic Beach at the Gulf of California, Mexico, International Journal of Environmental Health Research. (2025) 35, no. 11, 1–12, 10.1080/09603123.2025.2483356. 40131303

[48] Codesin , Radiografía del Turismo en Sinaloa Durante el Año 2022, 2023, Sinaloa, México, https://codesin.mx/file/9/139_PANORAMA%20ESTATAL%20DE%20TURISMO%20EN%20SINALOA%20-%202023.pdf_1700077596.pdf.

[49] Rubio-Lozano M. S. , Aspectos Reproductivos de la Raya *Urotrygon chilensis* (Günther, 1871) en las Costas de Sinaloa México, 2009, Universidad Nacional Autónoma de México, Ciudad de México, México, Bachelor’s thesis.

[50] Castellanos-Cendales A. L. , Hábitos Alimenticios de *Urobatis Halleri* y *Urotrigon chilensis* (Chondrichthyes: Urolophidae), En Sinaloa-México, 2009, Universidad Nacional Autónoma de México, Mazatlán, Sinaloa, México, Master’s thesis.

[51] Ohkubo N. , Ito M. , Hano T. , Kono K. , and Mochida K. , Estimation of the Uptake and Gut Retention of Microplastics in Juvenile Marine Fish: Mummichogs (*Fundulus heteroclitus*) and Red Seabreams (*Pagrus major*), Marine Pollution Bulletin. (2020) 160, 10.1016/j.marpolbul.2020.111630. 32911116

[52] Dean M. N. and Motta P. J. , Feeding Behavior and Kinematics of the Lesser Electric Ray, *Narcine brasiliensis* (Elasmobranchii: Batoidea), Zoology. (2004) 107, no. 3, 171–189, 10.1016/j.zool.2004.04.002. 16351936

[53] Garcés-García K. C. , Tovar-Ávila J. , Vargas-Trejo B. , Chávez-Arrenquín D. A. , Walker T. I. , and Day R. W. , Elasmobranch Bycatch by Prawn Trawls in the Gulf of California: First Comprehensive Analysis and the Effect of Fish Escape Devices, Fisheries Research. (2020) 230, 10.1016/j.fishres.2020.105639.

[54] O’Connor A. , Irhin K. , Sabo-Attwood T. , and Gray A. , Toxicological Impacts of Microplastic Fibers: A Review Assessing Risk to Human and Aquatic Health, Environmental Research. (2026) 288, no. Pt 2, 10.1016/j.envres.2025.123272. PMC12765480 41205791

[55] Mizukami-Murata S. , Tsushima I. , Abe S. , Kitamura T. , and Okayasu Y. , Effects of Laundry-Derived Microplastic Fibers on Larval Japanese Medaka (*Oryzias latipes*), The Science of the Total Environment. (2025) 983, 10.1016/j.scitotenv.2025.179657. 40398170

[56] Hu L. , Chernick M. , Lewis A. M. , Ferguson P. L. , and Hinton D. E. , Chronic Microfiber Exposure in Adult Japanese Medaka (*Oryzias latipes*), PLoS One. (2020) 15, no. 3, 10.1371/journal.pone.0229962. PMC7062270 32150587

[57] European Parliament and Council of the European Union , Regulation (EU) 2025/40 of 19 December 2024 on Packaging and Packaging Waste, 2024, European Parliament and Council of the European Union.

