# Supplementary material for: Anthropogenic Particle Ingestion in the Starry Round Ray Urotrygon asterias: High Occurrence Independent of Biological and Spatial Predictors
Source: Scientifica (Cairo). 2026 Oct 29;2026:1691340. doi: 10.1155/sci5/1691340 (PMC13717390; doi:10.1155/sci5/1691340)
Supplement: Supplementary file 1 [file SCI5-2026-1691340-s001.docx]

ST1. Anthropogenic particles detected in procedural blank controls during sample processing.

| Procedural blank | Material | Size (mm) |
| --- | --- | --- |
| Blank 2 | Cellulose | 2.018 |
| Blank 3 | Cellulose | 3.476 |
| Blank 7 | Polyester | 0.945 |
| Blank 9 | Cotton | 0.758 |
| Blank 26 | Cellulose | 1.456 |
| Blank 26 | Cellulose | 1.283 |

ST2. Positive-control fibers spiked into clean tissue and recovered after the complete digestion, filtration, and µFTIR-ATR workflow. All 12 fibers were recovered (100% recovery efficiency). Pre-spike material identities were known for all fibers. Dashes indicate fibers that were successfully recovered but did not yield a reliable µFTIR-ATR identification.

| **Fiber** | **Color** | **Pre-spike material identity** | **Post-recovery µFTIR identification** | **µFTIR match (%)** |
| --- | --- | --- | --- | --- |
| 1 | Black | Polyester (PET) | Polyester (PET) | 97 |
| 2 | Black | Cellulose | Not identified | — |
| 3 | Black | Cotton | Cotton | 68 |
| 4 | Black | Cellulose | Cellulose | 92 |
| 5 | Black | Cellulose | Not identified | — |
| 6 | Black | Cellulose | Cellulose | 81 |
| 7 | Black | Cellulose | Cellulose | 85 |
| 8 | Black | Rayon | Rayon | 89 |
| 9 | Blue | Cellulose | Cellulose | 83 |
| 10 | Blue | Cellulose | Cellulose | 93 |
| 11 | Blue | Cellulose | Cellulose | 91 |
| 12 | Blue | Cellulose | Not identified | — |

**ST3.** Comparison of count models and results of the negative binomial generalized linear model (GLM) of anthropogenic particle abundance in *Urotrygon asterias* (n = 114). (a) Comparison of the four candidate count models by log-likelihood, AIC and BIC; the negative binomial model, with the lowest AIC and BIC, was selected. (b) Estimated marginal means (± SE) per factor level, with sample sizes and Type II likelihood-ratio tests. (c) Slopes of the continuous predictors. No effect was statistically significant.

**(a) Model comparison (n = 114)**

| **Model** | **log-likelihood** | **AIC** | **BIC** |
| --- | --- | --- | --- |
| Poisson GLM | −237.57 | 489.13 | 508.28 |
| **Negative binomial GLM (selected)** | **−223.92** | **463.85** | **485.74** |
| Hurdle (binomial + zero-truncated NB) | −220.89 | 471.79 | 512.83 |
| Zero-inflated negative binomial | −223.92 | 465.85 | 490.47 |

*Likelihood-ratio test, Poisson vs negative binomial: χ² = 27.3, p < 0.001.*

**(b) Negative binomial GLM: estimated marginal means and effect tests**

| **Factor / level** | **Mean** | **SE** | **N** | **LR χ² (df)** | **p** |
| --- | --- | --- | --- | --- | --- |
| **Size class** |  |  |  | **1.15 (2)** | **0.562** |
| Small | 2.50 | 0.348 | 62 |  |  |
| Medium | 2.63 | 0.513 | 22 |  |  |
| Large | 1.93 | 0.407 | 30 |  |  |
| **Sex** |  |  |  | **0.15 (1)** | **0.701** |
| Female | 2.25 | 0.312 | 56 |  |  |
| Male | 2.45 | 0.337 | 58 |  |  |
| **Sampling zone** |  |  |  | **2.69 (2)** | **0.261** |
| Central | 2.04 | 0.244 | 65 |  |  |
| North | 2.51 | 0.565 | 16 |  |  |
| South | 2.98 | 0.567 | 33 |  |  |

**(c) Continuous predictors (negative binomial GLM)**

| **Predictor** | **Estimate (slope)** | **SE** | **p** |
| --- | --- | --- | --- |
| Dietary diversity (Shannon–Wiener index) | 0.517 | 0.408 | 0.206 |
| Disc width (cm) | −0.027 | 0.026 | 0.297 |

*Disc width was fitted in a separate model in which it replaced size class; all other predictors were unchanged.*

**ST4.** Comparison of anthropogenic particle contamination studies in Gulf of California elasmobranchs. The three-studies document widespread contamination across different taxa (batoids and sharks), trophic levels (benthic invertivores and apex predators), and sampling periods (2019-2020). Natural fiber dominance is a consistent regional signature. Occurrence rates vary (32-82%) due to methodological differences (natural fiber inclusion), temporal factors (season, year), and spatial factors (sampling locations). Only the *U. halleri* study (Pinho et al. 2022) collected environmental baseline data (sediment samples), highlighting the need for paired organism-environment sampling in future studies.

| Aspect | *U. asterias* | *U. halleri* (Baseline - Pinho et al. 2022) | *S. lewini* (Malthaner et al. 2024) |
| --- | --- | --- | --- |
| SPECIES | Starry round ray | Haller's round ray | Scalloped hammerhead shark (juvenile) |
| Taxonomic Group | Batoid (Urotrygonidae) | Batoid (Urotrygonidae) | Shark (Sphyrnidae) |
| Trophic Level | Benthic invertivore | Benthic invertivore | Piscivore/apex predator |
| Habitat | Sandy/muddy substrates, 5-50m | Sandy substrates, shallow coastal | Coastal/pelagic, nursery areas |
| SAMPLING | | | |
| Sample Size | 114 individuals | 34 individuals | 58 individuals |
| Sampling Period | July-August 2019 (2 months) | September-December 2020 (4 months) | Not specified (single collection) |
| Season | Summer | Fall (shrimp fishing season) | Unknown |
| Location | North, Central, South of Sinaloa | Eastern Gulf (Sonora coast) | Gulf of California (nursery areas) |
| Capture Method | Shrimp trawl bycatch | Shrimp trawl bycatch | Unknown (likely artisanal fisheries) |
| OCCURRENCE | | | |
| Occurrence Rate | 82.5% (94/114) | 32.4% (11/34) | 79.3% (46/58) |
| Mean Particles/Individual | 2.35 particles | Not clearly stated | Not clearly stated |
| Range | 0-12 particles | Not specified | Not specified |
| MATERIAL COMPOSITION | | | |
| Natural Fibers | 84.33% | Some cellulose-like materials (proportion unclear) | 64.4% cellulose |
| - Cellulose | 46.64% (125 particles) | Present but % not specified | 64.4% |
| - Cotton | 37.69% (101 particles) | Not specified separately | Not specified separately |
| Semisynthetic | 2.24% | Not specified | 3.2% Cellophane |
| -Rayon | 2.24% (6 particles) | Not specified | Not specified |
| Synthetic Polymers | 13.43% | Multiple types identified (nylon, PE, PP, polyacrylic) | Not specified (% synthetic unclear) |
| - Polyester | 10.07% (27 particles) | Present | Not specified |
| - Polypropylene | 1.12% (3 particles) | Present (prominent) | Not specified |
| - Other synthetics | 2.24% (acrylic, PAA, PEP, PU) | Nylon, PE, polyacrylic | Not specified |
| Total Particles Analyzed | 268 particles | Not clearly stated (multiple per individual) | Not clearly stated |
| ANALYTICAL METHODS | | | |
| Spectroscopy | µFTIR | FTIR | FTIR |
| Contamination Controls | Procedural blanks and positive controls included; blank contamination was quantified and low | Briefly mentioned, not quantitative | Not detailed |
| Detection Limit | Not explicitly stated (likely >0.5-1 mm) | Not explicitly stated | Not explicitly stated |
| PREDICTORS TESTED | | | |
| Sex | No significant effect on particle abundance (Negative binomial GLM) or particle composition (PERMANOVA). | Not tested or reported | Not tested or reported |
| Body Size | No significant effect on particle abundance (negative binomial GLM) or particle composition (PERMANOVA); no significant relationship between disc width and particle abundance (Spearman correlation). | Not tested or reported | Not tested or reported |
| Diet | Dietary diversity was not significantly associated with particle abundance (Negative binomial GLM), and fitting anthropogenic particle types as vectors onto the Principal Coordinates Analysis (PCO) revealed no clear association with diet composition. | Not tested | Not applicable (different feeding mode) |
| Spatial Zone | No significant effect on particle abundance (negative binomial GLM) or particle composition (PERMANOVA). However, multivariate dispersion differed significantly among zones (PERMDISP). | Not tested (single location) | Not tested or reported |
| KEY FINDING | Anthropogenic particles were frequently detected, with no significant effects of biological or spatial predictors on particle abundance or multivariate composition. However, within-zone variability in particle composition differed among capture zones, and no clear association between dietary patterns and particle ingestion was observed. | Contamination present in benthic batoid; sediment data provided | Very high occurrence in apex predator juveniles; cellulose dominance |
| ENVIRONMENTAL DATA | | | |
| Sediment Samples | NOT COLLECTED | YES - 12-156 particles/kg dry weight | NOT COLLECTED |
| Water Samples | NOT COLLECTED | Not specified | NOT COLLECTED |
| Sediment Fiber Dominance | N/A | YES - fibers dominant morphology | N/A |
| LIMITATIONS | | | |
| Temporal Coverage | Single season (2 months) | Single season (4 months) | Unknown temporal coverage |
| Environmental Baseline | ABSENT | PRESENT | ABSENT |
| Sample Size | Adequate (n=114) | Small (n=34) | Adequate (n=58) |
| Spatial Coverage | Broad (3 zones) | Limited (single region) | Unknown |
| CONSERVATION STATUS | | | |
| Bycatch Vulnerability | HIGH (all specimens from bycatch) | HIGH (all specimens from bycatch) | MODERATE-HIGH (targeted + bycatch) |
| Conservation Concern | Data Deficient (likely) | Data Deficient | Critically Endangered (IUCN) |
